# SpirPep: an in silico digestion-based platform to assist bioactive peptides discovery from a genome-wide database

**DOI:** 10.1186/s12859-018-2143-0

**Published:** 2018-04-20

**Authors:** Krittima Anekthanakul, Apiradee Hongsthong, Jittisak Senachak, Marasri Ruengjitchatchawalya

**Affiliations:** 1 Biotechnology Program, School of Bioresources and Technology, King Mongkut’s University of Technology Thonburi (Bang Khun Thian), 49 Soi Thian Thale 25, Bang Khun Thian Chai Thale Rd., Tha Kham, Bang Khun Thian, Bangkok, 10150 Thailand; 2 Biochemical Engineering and Pilot Plant Research and Development Unit, National Center for Genetic Engineering and Biotechnology at King Mongkut’s University of Technology Thonburi, 49 Soi Thian Thale 25, Bang Khun Thian Chai Thale Rd., Tha Kham, Bang Khun Thian, Bangkok, 10150 Thailand; 3 Bioinformatics and Systems Biology Program, School of Bioresources and Technology, King Mongkut’s University of Technology Thonburi (Bang Khun Thian), 49 Soi Thian Thale 25, Bang Khun Thian Chai Thale Rd., Tha Kham, Bang Khun Thian, Bangkok, 10150 Thailand

**Keywords:** SpirPep, Genome, Bioactive peptides, In silico, Bioactive peptide discovery, GBrowse

## Abstract

**Background:**

Bioactive peptides, including biological sources-derived peptides with different biological activities, are protein fragments that influence the functions or conditions of organisms, in particular humans and animals. Conventional methods of identifying bioactive peptides are time-consuming and costly. To quicken the processes, several bioinformatics tools are recently used to facilitate screening of the potential peptides prior their activity assessment in vitro and/or in vivo. In this study, we developed an efficient computational method, SpirPep, which offers many advantages over the currently available tools.

**Results:**

The SpirPep web application tool is a one-stop analysis and visualization facility to assist bioactive peptide discovery. The tool is equipped with 15 customized enzymes and 1–3 miscleavage options, which allows in silico digestion of protein sequences encoded by protein-coding genes from single, multiple, or genome-wide scaling, and then directly classifies the peptides by bioactivity using an in-house database that contains bioactive peptides collected from 13 public databases. With this tool, the resulting peptides are categorized by each selected enzyme, and shown in a tabular format where the peptide sequences can be tracked back to their original proteins. The developed tool and webpages are coded in PHP and HTML with CSS/JavaScript. Moreover, the tool allows protein-peptide alignment visualization by Generic Genome Browser (GBrowse) to display the region and details of the proteins and peptides within each parameter, while considering digestion design for the desirable bioactivity. SpirPep is efficient; it takes less than 20 min to digest 3000 proteins (751,860 amino acids) with 15 enzymes and three miscleavages for each enzyme, and only a few seconds for single enzyme digestion. Obviously, the tool identified more bioactive peptides than that of the benchmarked tool; an example of validated pentapeptide (FLPIL) from LC-MS/MS was demonstrated. The web and database server are available at http://spirpepapp.sbi.kmutt.ac.th.

**Conclusion:**

SpirPep, a web-based bioactive peptide discovery application, is an in silico-based tool with an overview of the results. The platform is a one-stop analysis and visualization facility; and offers advantages over the currently available tools. This tool may be useful for further bioactivity analysis and the quantitative discovery of desirable peptides.

**Electronic supplementary material:**

The online version of this article (10.1186/s12859-018-2143-0) contains supplementary material, which is available to authorized users.

## Background

Bioactive peptides (BP) are protein fragments or peptides that play a significant role in human and animal health [1] and can be classified as endogenous; natural synthesis in the organisms or exogenous; by food-derived digestive processes using gastrointestinal (GI) enzymes in the GI-tract [2, 3]. These peptides can be found in many sources, such as dairy products [4], land animals [5], marine animals [6, 7], plants [8] and cyanobacteria [9]. The bioactivities of these peptides are classified according to their primary structure and ability to form products. The majority of peptides derived from food proteins are generated by enzymatic hydrolysis. Examples include “IQP”, an antihypertensive peptide from *Spirulina platensis* after digestion with alcalase from *Bacillus licheniformis* [10], and “YAEERYPIL”, an angiotensin I-converting enzyme (ACE) inhibitor and antioxidant peptide from the hydrolysate of ovalbumin with pepsin [11]*.* Although, enzymatic hydrolysis is a preferred method, several peptide-containing food products available in markets are generated from microbial fermentation. For example, sour milk and fermented milk which contain lactotripeptides such as isoleucine-proline-proline (IPP) and valine-proline-proline (VPP) [12, 13], were reported by European Food Safety Authority (EFSA) to have no significant effect in maintaining normal blood pressure [14]. However, other reports based on meta-analysis reported that IPP and VPP lactotripeptides could significantly reduce systolic blood pressure in Japanese subjects [13, 15].

Conventionally, the discovery of bioactive peptides is a time-consuming and costly process. The classical approach consists of enzyme selection, peptide production from protein hydrolysis, peptide-purification, peptide-identification, and in vivo or in vitro assays. Recently, the use of computational tools offers relief as they shorten time for the screening of peptide candidate prior to purification and the biochemical validation process in the laboratory. Currently available online tools can be classified into three groups: (i) in silico peptide digestion tools, e.g., PeptideCutter, a tool with a single protein sequence input, various enzymes and chemical options, can generate peptide sequences, allowing users to manually search for bioactivities against other databases [16], (ii) bioactive peptide prediction tools, e.g., PeptideLocator that directly predicts possible bioactive peptide sequence locations in protein sequences from the input of UniProt IDs [17] and PeptideRanker, which computes the bioactive peptide probability from input peptide sequence [18], and (iii) tools that combine in silico digestion and bioactive peptide prediction, e.g., mMass and BIOPEP tools. The mMass tool allows only one protein sequence and one enzyme as inputs, with an option of miscleavage [19], while BIOPEP tool allows one protein sequence and up to three enzymes as inputs, with an option to search against one bioactive peptide database [20]. Practically, a protein is hydrolyzed with a protease at the cleavage sites accord with cleavage rules, mentioned as ‘no miscleavage’. However, there is often found ‘miscleavage’, a lack of cleavage by the designated protease at one or both ends of the peptide, but cleavage at the other, resulting from an incomplete protein digestion (see Additional file 1: Figure S1) based on factors including protein structure, digestion technique, source of enzyme and enzyme kinetics [21–24]. The cleavable site(s) can be skipped due to configuration and sequence of amino acid residues leading to sterically inaccessible for the enzyme and/or slow kinetics [22], as example of Keil rule for miacleavage by trypsin [25].

Due to the advantages of bioinformatics tools, the in silico methodology is used for in silico peptide digestion and predicting bioactive peptides; for example, dipeptidyl peptidase-IV and angiotensin I-converting enzyme inhibitory peptides were identified by combining online in silico tools (BIOPEP, PeptideCutter tool and PeptideRanker) as an in-house tool [26]. However, these tools are incapable of the input of multiple protein sequences and have no miscleavage option.

In this work, we, therefore, develop a web-based application tool, SpirPep, which is a one-stop analysis and visualization pipeline for bioactive peptide discovery. Our pipeline is able to rapidly predict putative peptides by the in silico digestion of protein(s) up to a genome-wide level with the choice of 15 restriction enzymes and the number of miscleavages as input parameters. The resulting peptides are then searched against our in-house database collected from 13 public bioactive peptide databases for bioactive peptide identification. All peptides are kept temporarily in the database and are used to visualize protein-peptide alignment and display the region as well as details of the proteins and peptides listed separately for each input parameter by the Generic Genome Browser (GBrowse) [27]. In addition, we developed the GBrowse-based visualizer to generate a location overview of the bioactive peptides on their original protein, thus, it could provide potential decision-making information to assist users in re-designing the digestive systems, of which enzymes, miscleavages and proteins are considered to achieve the desirable bioactive peptide. The key performance comparison of the bioactive peptide identification tools is shown in detail in Table 1. Therefore, our SpirPep provides a shortcut for efficient screening and identifying target bioactive peptides and is applicable for other organisms of interest.

**Table 1:** Key performance comparison of ‘SpirPep’ to other bioactive peptide identification tools

Category	Bioactive peptide identification tool	Input	In silico peptide digestion		Bioactive peptide prediction			Result of in silico peptide digestion/bioactive peptide prediction tool			Accessibility
					Bioactivity		No. of known bioactive peptide sequence				
		No. of protein per analysis	No. of Enzyme selection	No. of Miscleavage	Yes/No	Identification		Original protein track back	Resulting peptide categorized by enzyme	Protein-peptide alignment visualization by GBrowse	
In silico peptide digestion tool	PeptideCutter tool	1 protein	1–29	N/A	No	N/A	N/A	N/A	√	N/A	http://web.expasy.org/peptide_cutter/
Bioactive peptide prediction tool	PeptideLocator	1 Uniprot ID (≤10,000 aa)	N/A	N/A	Yes	N/A	Unknown	N/A	N/A	N/A	http://bioware.ucd.ie/~compass/biowareweb/Server_pages/biopred.php
	PeptideRanker	Up to 150 peptides	N/A	N/A	Yes	N/A	Unknown	N/A	N/A	N/A	http://bioware.ucd.ie/~compass/biowareweb/Server_pages/peptideranker.php
In silico peptide digestion and bioactive peptide prediction tool	mMass	1 protein	1	As user input	Yes	√	612 sequences	N/A	N/A	N/A	Standalone version (http://www.mmass.org/download/)
	BIOPEP	1 protein	1–3	N/A	Yes	√	3587 sequences	N/A	N/A	N/A	http://www.uwm.edu.pl/biochemia/index.php/en/biopep
	SpirPep (In this study)	Thousands proteins or the whole genome	1–15	0–3	Yes	√	28,892 sequences	√	√	√	http://spirpepapp.sbi.kmutt.ac.th/SpirPep/SpirPepTool

√ = Available; N/A = Not available

## Construction and content

SpirPep was constructed as a web-based application tool for bioactive peptide discovery by the in silico peptide digestion of protein sequences derived from protein-coding genes such as single sequences, multiple sequences, and genome-wide scales. This tool consists of a database server and a web server for bioactive peptide discovery.

### Databases

**Fig. 1:**
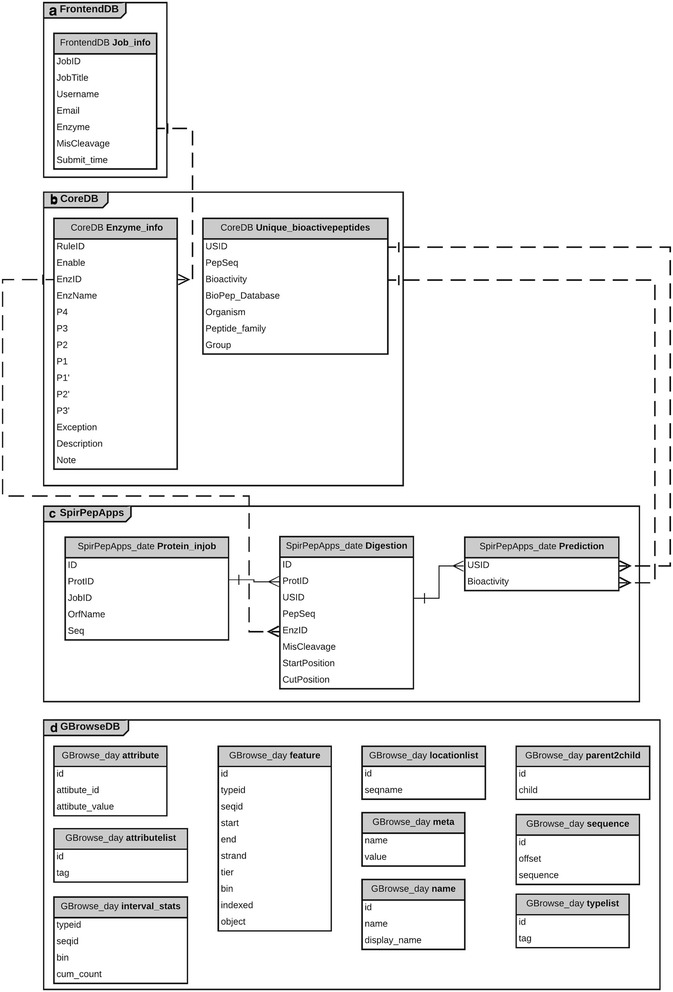
Database schematic diagram for all databases in the SpirPep web application: **a** FrontendDB, **b** CoreDB, **c** SpirPepApps and **d** GBrowseDB

Four databases were constructed for storing data and putative peptides from each process as well as to integrate the in silico peptide results with bioactive peptide sequences retrieved from 13 public bioactive peptide databases, which contained both naturally synthesized and hydrolysed proteins (Table 2 and Fig. 1). The peptides were screened for bioactivity redundancy, thereby resulting in bioactive peptides with single or multi-functional bioactivity whose information source could be tracked. The first database, FrontendDB, stores queries from user input, which consists of user information, job title, enzyme and miscleavage of choice, and statistical analysis time (Fig. 1a). The second, CoreDB, contains the restriction site rules of the enzymes (by SpirPep default, 15 enzymes from the PeptideCutter tool [16]) and details the non-redundant bioactive peptide sequences from the retrieved bioactive peptide databases (Fig. 1b). The third, SpirPepApps, stores the input protein sequences and the putative peptide sequences from in silico peptide digestion, non-redundant peptide sequences, and the exact matching results from the comparison with retrieved bioactive peptide sequences (Fig. 1c). The fourth, GBrowseDB, stores the data generated in the GFF file format containing the matched results for protein-peptide alignment visualization (Fig. 1d).

**Table 2:** Names and descriptions of online bioactive peptide databases (Accessed 11 January 2018)

No.	Database name	Biological Function	Database Description	Last Update	Reference
1.	APD	Antimicrobial	Antimicrobial and anticancer peptides	Jan 2018	[31]
2.	BACTIBASE	Antibacterial	Antibacterial peptides (Bacteriocins)	May 2017	[32]
3.	BAGEL3	Antibacterial	Antibacterial peptides (Bacteriocins)	^a^/2018	[33]
4.	CAMP	Antimicrobial	Antimicrobial peptides and proteins	Apr 2010^b^	[34]
5.	Defensins knowledgebase	Defensin, Antimicrobial	Antimicrobial peptides from the defensin family	Aug 2010^b^	[35]
6.	EROP-Moscow	-	Endogenous Regulatory oligopeptides	Nov 2016	[36]
7.	Hmrbase	Hormone	Hormones & receptors	May 2009	[37]
8.	PenBase	Antimicrobial	Database of antimicrobial peptides from penaeid shrimps	Now not available (Jul 2008)	[38]
9.	PeptideDB	Various bioactivities	Biologically active peptides, peptide precursors and motifs in Metazoa.	Apr 2008	[39]
10.	PhytAMP	Antimicrobial	Antimicrobial peptides and proteins of plant origin	Jan 2012	[40]
11.	RAPD	Antimicrobial	Recombinant antimicrobial peptides	Now not available (Mar 2010)	[41]
12.	ACEpepDB	Antihypertensive	Food derived antihypertensive peptides that are available in the literature	Now not available (^a^/2011)	[42]
13.	BIOPEP	Various bioactivities	Food proteins	^a^/2008^b^	[20]

^a^represents an unavailable month

^b^The database is updated but not shown the date on the website

To manage input and output data, we created both SpirPepApps and GBrowseDB by date and deleted the entire database information within 3 days due to storage space limitation.

### SpirPep web application

**Fig. 2:**
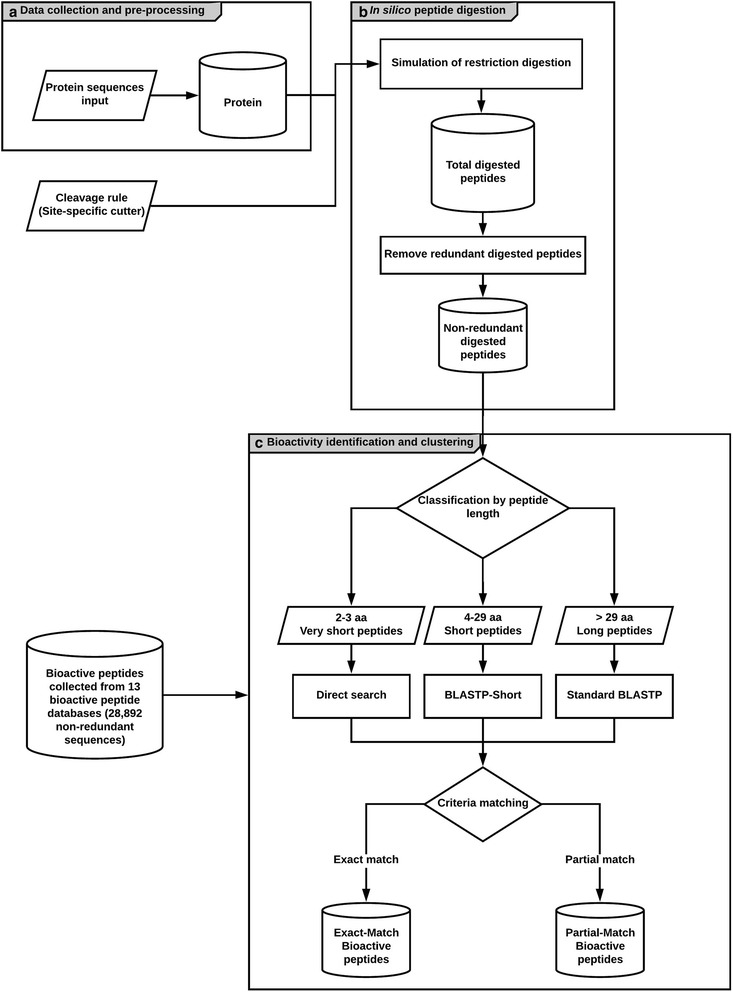
SpirPep workflow: This workflow is based on the in silico peptide digestion for bioactive peptide discovery. There were three modules: **a** data collection and pre-processing: the protein sequences were sent to the Protein database; **b** in silico peptide digestion: protein sequences were digested with the selected enzymes and the miscleavage number and non-redundant digested peptides were removed to classify them into three groups by peptide length (very short, short, and long peptides); and **c** bioactivity identification and clustering where these peptide groups were compared against the bioactive peptide sequences with the different methods with 100% in both identity and query or subject coverage

The SpirPep web-based application tool has been developed to facilitate the use of SpirPep workflow (Fig. 2). It is useful for discovering bioactive peptides from protein-coding genes in genomes by in silico peptide digestion. It is a three-tier system containing front-end (left), queuing system (middle), and back-end (right) sites, as shown in the sequence diagram (see Additional file 2: Figure S2). The front-end site, which is written in PHP, accepts input queries from users, then encapsulates the user’s queries into jobs, and sends them through the back-end site. Using the PHP-Resque queuing framework on the Redis server [28], each Resque worker on the back-end server takes the job from the queue and proceeds into the SpirPep workflow.

First, it invokes the in silico peptide digestion module with the given proteins and parameters (by default, using the enzyme trypsin with no miscleavage allowed). Then, all non-redundant digested peptides are compared against the in-house bioactive peptide database, which is gathered from both naturally synthesized and hydrolysed proteins (Table 2). The matched results are then generated in the GFF file format for protein-peptide alignment visualization. The completed results are next sent back to the front end in order to send a notification email to users with link to the results page. In the results page, the exact matched data, bioactive peptide sequences, and protein-peptide alignment visualization from SpirPepApps, CoreDB, and GBrowseDB databases, respectively, are retrieved (Additional file 2: Figure S2).

## Utility

The SpirPep web application contains five parts: Home, SpirPep Tool, Bioactive Peptide Database, User Guide, and Contact Us.

***Home***; presents an introduction to bioactive peptide discovery, motivation of the computational method for bioactive peptide prediction and advantages of the SpirPep web application.

***SpirPep Tool***; here the user can input multiple protein sequences of interest, whole organism genome, or upload files in FASTA format with the required parameters. User replies are sent via email and displayed in the response page. Afterwards, SpirPep Tool predicts the candidate bioactive peptide sequences and then sends the output to the user by email.

***Bioactive Peptide Database***; stores the number and biological function of retrieved bioactive peptides from 13 online bioactive peptide databases and their respective tree maps, grouped by size (very short peptides with di- and tri-peptides, short peptides with 4–29 amino acids, and long peptides with more than 29 amino acids).

***User Guide***; assists user’s to familiarize themselves with the contents and functionalities embedded in the *SpirPep Tool*. It demonstrates the processes involved from inputting queries into SpirPep Tool and accessing the results from the email notification. Additional file 3: Figure S3 shows an example of all of the steps, which can be divided into three major parts: (i) “Query input” for inputting three protein sequences with the trypsin enzyme and the miscleavage limit set to one miscleavage (Additional file 3: Figure S3a); (ii) “Notification” after the submission as a response page and email with the submission information and email notification when the results are completed with the link to the two results pages (Additional file 3: Figure S3b); and (iii) “Result pages” for showing the screening results of candidate bioactive peptides from proteins and selected parameters (Additional file 3: Figure S3c). On the results page, the “List all” provides details of the bioactive peptides from all of the digested proteins. The example shows three di-bioactive peptide sequences (FK, QK and KK) from protein SPLC1_S010010 digested with the trypsin enzyme with zero and one miscleavage. These peptide sequences can be tracked back to their original proteins separated by a comma (,). The “Summary” presents the list and number of predicted bioactive peptides within the proteins sequences. The number can be linked to the bioactive peptide sequence information, as derived from bioactive peptide databases and organisms. The visualization page in GBrowse tool shows the position of the predicted bioactive peptides on an individual protein. The user-interface feature displays three graphical panels namely “Overview”, “Region,” and “Detail” providing access to the regions and detailed overview of the protein and peptide alignment. In the “Detail” view, the protein and derived peptide sequences from individual enzymes and miscleavages are organized into the “Protein” and “Enzyme name” tracks, respectively. The “Enzyme name” tracks are organized with different colours for convenient viewing, while an individual is divided into the miscleavage number as subtracks. The features are organized as glyphs with tracks and subtracks that display all of the peptide sequences with bioactivity as popup balloon tooltips. For this example, three derived peptide sequences (FK and QK from zero miscleavage and KK from one miscleavage) of SPLC1_S010010 protein are aligned on the “trypsin enzyme track” with the deep sky-blue colour and defined with the miscleavage number subtracks (Additional file 3: Figure S3c). In the case where more than one enzyme with the same protein sequence is produced, users can overview all enzyme tracks by selecting the enzyme(s) on “Select Tracks” to show the optional track in the “Browser”. The region can be scrolled and zoomed with the buttons in the header for overviewing the available peptide sequences along the proteins. For viewing all enzymes, users can consider re-designing the single digestion to double digestion to retrieve more desirable bioactive peptide sequences. The results are presented in tabular format, which users can copy, export as Excel, CSV, or PDF files, or even print by clicking the buttons in the table header and then search for the desired data and filter the results. Our bioinformatics tool allows users to quickly screen candidate bioactive peptides from a set of proteins before validation in laboratory.

***Contact Us***; provides the contact information of the SpirPep Team from KMUTT for consulting, suggestions, or usage problems.

## Discussion

In comparison to other available bioactive peptide prediction tools (Table 1), SpirPep contains several unique features that differ from the online tools mentioned above. First, it has the capability for completing the entire process of in silico digestion and bioactive peptide prediction from multiple protein sequences or whole genome input, desirable enzyme selection with the miscleavage option and searching against in-house bioactive peptide databases (28,892 sequences) for bioactive peptide identification. Second, SpirPep output allows back-tracking of the resulting peptides to their original proteins, categorizes them by enzyme and miscleavage parameters, and performs protein-peptide alignment visualization by GBrowse, which presents alignment overview of derived peptide sequences on their original protein separately, based on the selected parameters with the bioactivity popup balloon tooltips and also helps in re-designing digestion for double digestion to meet user’s research needs. Moreover, the entire process of SpirPep takes less than 20 min for the digestion of 3000 proteins (751,860 amino acids) with 15 enzymes and three miscleavages for each enzyme or only a few seconds for single enzyme digestion. However, in silico peptide digestion tools are protein digestion simulations with selected parameters that may be different from in vitro digestion. From the structure of the Enzyme_info table of the CoreDB database (Fig. 1), we can easily modify the restriction site rules of enzymes that are commercially available and also add more rules for other enzymes. Our ongoing study focuses on the discovery of novel bioactive peptides by in vitro digestion using the enzyme obtained from SpirPep prediction which yields the peptides of interest.

For benchmarking, we compared SpirPep to another tool classified in the same category, BIOPEP (Table 1), using the same protein dataset, and selecting trypsin enzyme with no miscleavage allowed. Results showed the number of bioactive peptides identified by SpirPep were more than that of BIOPEP, although the generated peptides are less (Table 3 and Additional file 4: Table S1). The difference in the number of generated peptide sequences of each tool is in accord with the different cleavage rules. Cleavage sites of trypsin in ‘BIOPEP’ tool are at the C-terminal of lysine or arginine residues, whereas the cleavage rules in ‘SpirPep’ are referred from the PeptideCutter tool. In our tool, cleavage sites of trypsin are at the C-terminal of lysine or arginine residues with no proline at the C-terminal of lysine or arginine. However, this blocking of cleavage exerted by proline is negligible when methionine is at the N-terminal of arginine or tryptophan at the N-terminal of lysine with some exceptions. SpirPep identified more bioactive peptides on account of the ‘miscleavage’ option and the capacity of our in-house bioactive peptide database as shown in Tables 3 and 4. The generated peptides from BIOPEP were annotated with our in-house bioactive peptide database resulting in a higher number of identified bioactive peptides compared to that of BIOPEP, which demonstrates the capacity of our in-house bioactive peptide database. However, we found that prediction success was also dependent on the selected enzyme, for example, no bioactive peptide was found by trypsin; whereas for thermolysin digestion, bioactive peptides were found (Table 4).

**Table 3:** Output comparison between SpirPep and BIOPEP, the identified bioactive peptides obtained from temperature stress – expressed protein [30] digested with trypsin and no miscleavage

In silico peptide digestion tool	No. of peptide	No. of bioactive peptide (Percentage of No. of peptides)	
		SpirPep	BIOPEP
SpirPep	4333	418 (9.65)	371 (8.56)
BIOPEP	4556	450 (9.88)	399 (8.76)

**Table 4:** Output comparison of identified bioactive peptides of genomic proteins of *Arthrospira platensis* strain C1 from SpirPep by selecting trypsin and thermolysin enzyme with miscleavage allowed up to three miscleavages against SpirPep in-house bioactive peptide and BIOPEP

Selected enzymes	Miscleavage option											
	0			1			2			3		
	No. of peptide	No. of bioactive peptide^a^		No. of peptide	No. of bioactive peptide^a^		No. of peptide	No. of bioactive peptide^a^		No. of peptide	No. of bioactive peptide^a^	
		SpirPep	BIOPEP		SpirPep	BIOPEP		SpirPep	BIOPEP		SpirPep	BIOPEP
Trypsin	99,905	298 (0.3%)	247 (0.25%)	135,899	48 (0.04%)	39 (0.03%)	137,309	1 (0%)	1 (0%)	133,145	0 (0%)	0 (0%)
Thermolysin	115,846	302 (0.26%)	249 (0.21%)	285,983	182 (0.06%)	140 (0.05%)	403,052	56 (0.01%)	32 (0.01%)	449,652	15 (0%)	10 (0%)

^a^ The number in bracket is the percentage of bioactive peptide

Additionally, in our testing (unpublished data), 6108 *Spirulina* proteins were input into ‘SpirPep’, the enzyme thermolysin was selected, and up to three miscleavage was allowed (see example of our obtained result in Additional file 1: Figure S1b). The output showed that 1,379,992 peptides were obtained from three groups of peptides (very short, short and long peptides) with 572 bioactive peptides (0.04% of the total peptides), of 2–7 amino acids peptide length. For validation, the protein extracted from *Spirulina* cells grown at optimal condition was in vitro digested with thermolysin enzyme and sequentially isolated and analyzed by Liquid chromatography-tandem mass spectrometry (LC-MS/MS, Dionex UltiMate™ 3000 RSLCnano System (Thermo Fisher Scientific, Waltham, MA, USA) and MaXis II Mass Spectrometer Detector (Bruker, Germany)). The identified results from LC-MS/MS contained 3372 proteins (55.21% of the total genomic proteins) according to protein expression and 7366 peptides (0.53% of in silico peptides). The sample digested by thermolysin prior to LC-MS/MS were 6233 in common with the peptides found in the in silico ‘SpirPep’. Due to the limitation of LC-MS/MS detection, there are the peptides predicted by SpirPep but not present in the results from LC-MS/MS, which consist of only 5–24 amino acids peptide length. Hence, we could find only 76 (5–7 amino acids peptide length), but lost 496 (including di-, tri- and tetrapeptides) bioactive peptides. Fortunately, a pentapeptide (FLPIL, with a neuropeptide property) from LC-MS/MS (0.17% of the total in silico bioactive peptides, 572) was also detected in ‘SpirPep’ (1.32% of the in silico found, 76), but not in ‘BIOPEP’ (see additional related information (Table 3) for the output comparison between ‘SpirPep’ and ‘BIOPEP’).

## Conclusions

SpirPep is a one-stop web application that offers rapid identification and efficient analysis of bioactive peptides with six key features: (i) genome-wide scale inputs, (ii) a miscleavage option, (iii) the output of a number of known bioactive peptides for bioactivity identification, (iv) the resulting peptide categorized by enzyme, (v) the original protein tracked, and (vi) a GBrowse-based visualizer. Hence, SpirPep is a promising alternative pipeline for efficient screening and identification of bioactive peptides and their proteins of origin.

## Availability and requirements

Project name: SpirPep: A web-based database for bioactive peptide discovery. Project home page: http://spirpepapp.sbi.kmutt.ac.th. Operation system(s): Web based, Platform independent. Programming language: HTML, CSS, JavaScript, MySQL, PHP.

## Additional files

Additional file 1: **Figure S1.** Schematic diagram of miscleavage. a) Example of miscleavage occurrence, b) Example of peptides resulting of thermolysate of molecular chaperone DnaK (SPLC1_S010870) from LC-MS/MS at amino acid 1–26 and 123–134 (Our unpublished data). (TIFF 8492 kb)

Additional file 2: **Figure S2.** SpirPep sequence diagram: SpirPep was designed into a three-tier system (front-end, queuing, and back-end). The front-end sends the queries to SpirPepDB (FrontendDB and SpirPepApps) and responds to the users. The queries are queued by the Redis server and sent to Resque worker(s) in the back-end. When the analysis is complete, the system will send an email notification with the link to the results page to users. The results will be stored in the database temporary table and exported to the GFF file format, which can be internally used by the SpirPep visualizer. (JPEG 466 kb)

Additional file 3: **Figure S3.** Example of webpage snapshots from the SpirPep tool: a) query input from user submission (protein sequences, user’s name and email, job title, desired enzyme(s) and allowed miscleavage number), b) notification of received query from users as the response page and submission information email when the analysis complete, and c) results pages that contain the list all and summary pages, which show the predicted bioactive peptide sequences derived from input proteins and parameters. This provides a valuable decision to users for their re-designing the digestion system to obtain desirable bioactive peptides and enzymes before validation in a laboratory. (JPEG 2333 kb)

Additional file 4: **Table S1.** Dataset and output comparison between SpirPep and BIOPEP, the identified bioactive peptides obtained from temperature stress – expressed protein [30] digested with trypsin and no miscleavage. (XLSX 18 kb)

## Abbreviations

CSS: Cascading style sheet
CSV: Comma separated values
GBrowse: Generic genome browser
HTML: Hypertext markup language
MySQL: My structured query language
PDF: Portable document format
PHP: Hypertext preprocessor
URL: Uniform resource identifier
