# Supplementary figures and images for: SpirPep: an in silico digestion-based platform to assist bioactive peptides discovery from a genome-wide database
Source: BMC Bioinformatics. 2018 Apr 20;19:149. doi: 10.1186/s12859-018-2143-0 (PMC5910554; doi:10.1186/s12859-018-2143-0)

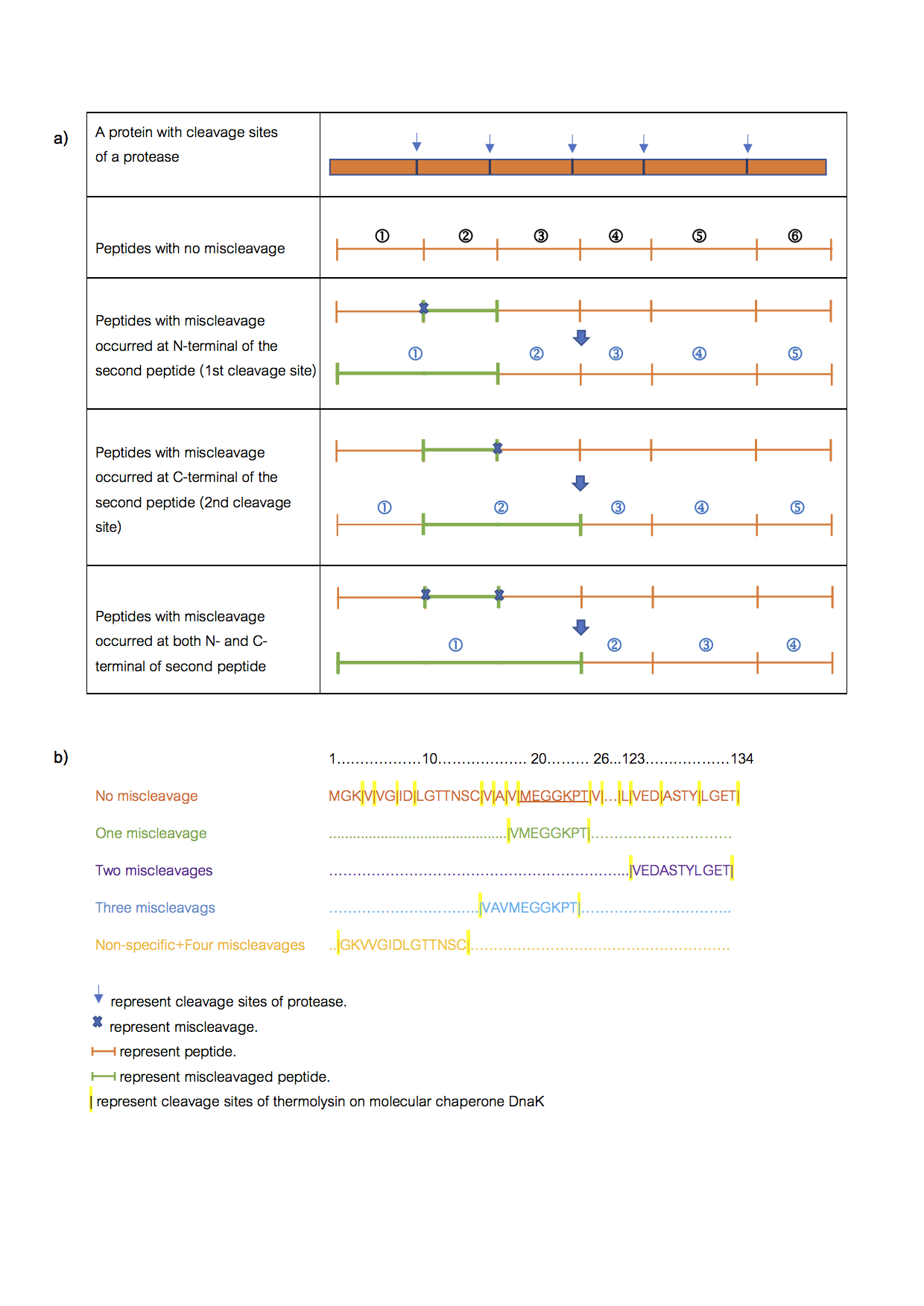

Supplement: Supplementary file 1 — Figure S1. Schematic diagram of miscleavage. a) Example of miscleavage occurrence, b) Example of peptides resulting of thermolysate of molecular chaperone DnaK (SPLC1_S010870) from LC-MS/MS at amino acid 1–26 and 123–134 (Our unpublished data). (TIFF 8492 kb) [file 12859_2018_2143_MOESM1_ESM.tiff]

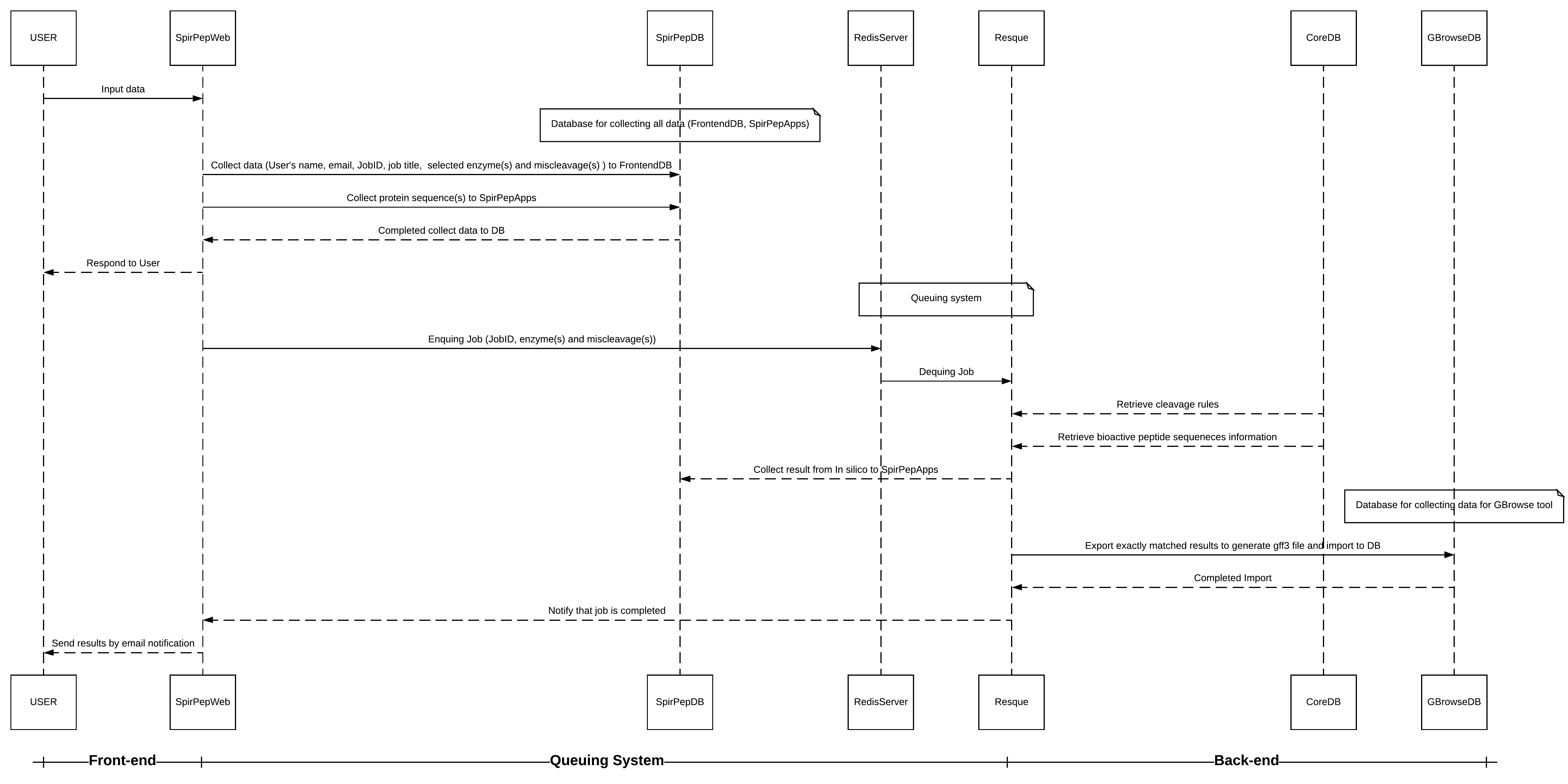

Supplement: Supplementary file 2 — Figure S2. SpirPep sequence diagram: SpirPep was designed into a three-tier system (front-end, queuing, and back-end). The front-end sends the queries to SpirPepDB (FrontendDB and SpirPepApps) and responds to the users. The queries are queued by the Redis server and sent to Resque worker(s) in the back-end. When the analysis is complete, the system will send an email notification with the link to the results page to users. The results will be stored in the database temporary table and exported to the GFF file format, which can be internally used by the SpirPep visualizer. (JPEG 466 kb) [file 12859_2018_2143_MOESM2_ESM.jpeg]

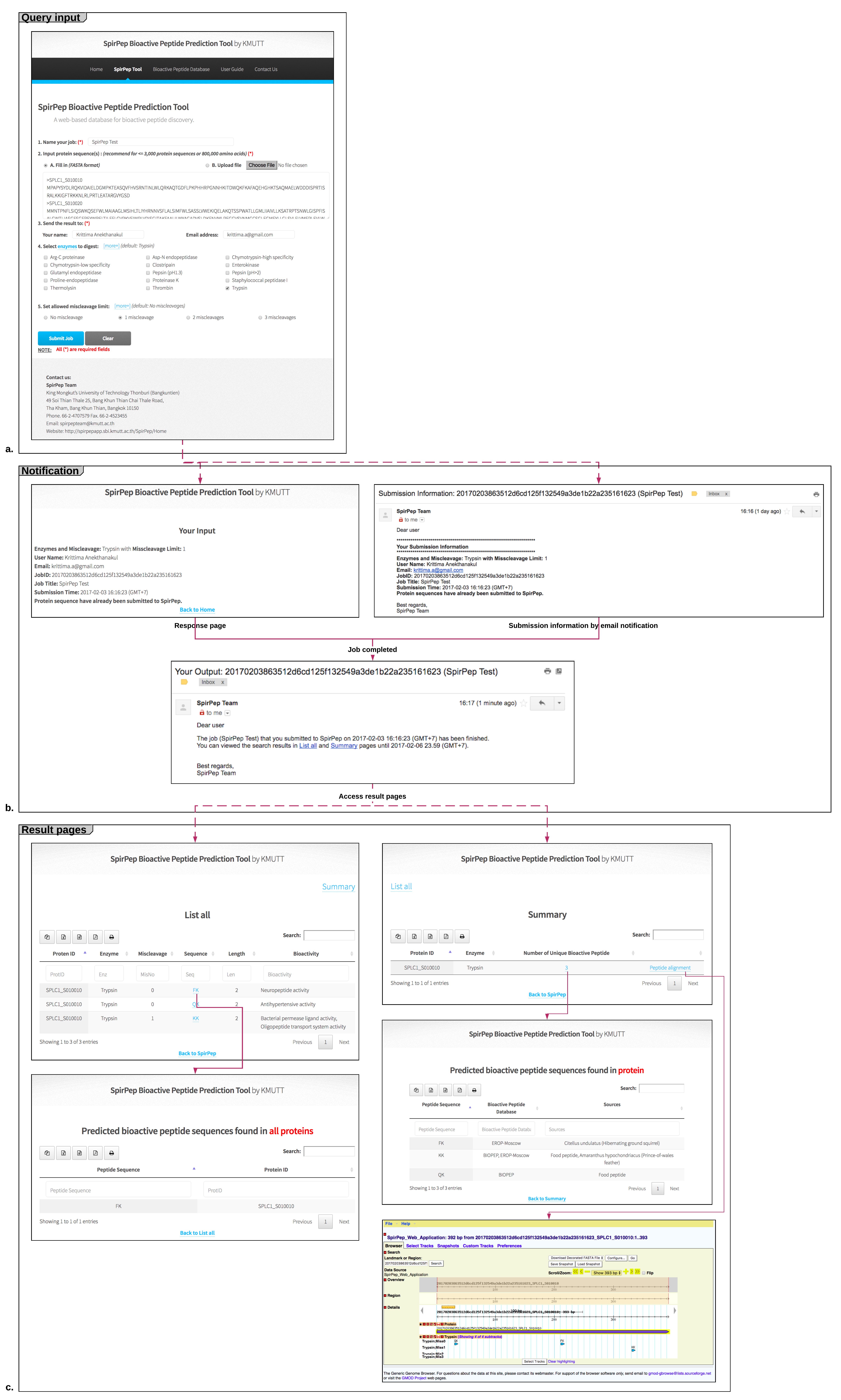

Supplement: Supplementary file 3 — Figure S3. Example of webpage snapshots from the SpirPep tool: a) query input from user submission (protein sequences, user’s name and email, job title, desired enzyme(s) and allowed miscleavage number), b) notification of received query from users as the response page and submission information email when the analysis complete, and c) results pages that contain the list all and summary pages, which show the predicted bioactive peptide sequences derived from input proteins and parameters. This provides a valuable decision to users for their re-designing the digestion system to obtain desirable bioactive peptides and enzymes before validation in a laboratory. (JPEG 2333 kb) [file 12859_2018_2143_MOESM3_ESM.jpeg]
